# Morphological and immunohistochemical evaluation in distinguishing post-radiotherapy serous-like endometrial change (PoRSEC) and serous endometrial intraepithelial carcinoma (SEIC)

**DOI:** 10.1007/s00428-024-03818-4

**Published:** 2024-07-30

**Authors:** Damiano Arciuolo, Giulia Scaglione, Antonio Travaglino, Nicoletta D’Alessandris, Angela Santoro, Frediano Inzani, Belen Padial Urtueta, Stefania Sfregola, Antonio Raffone, Caterina Fulgione, Michele Valente, Roberta Benvenuto, Federica Cianfrini, Gian Franco Zannoni

**Affiliations:** 1 Gynecopathology and Breast Pathology Unit, Department of Woman and Child’s Health and Public Health Sciences, Fondazione Policlinico Universitario Agostino Gemelli IRCCS, 00168 Rome, Italy; 2 Pathology Unit, Department of Medicine and Technological Innovation, University of Insubria, Varese, Italy; 3 Department of Medical and Surgical Sciences (DIMEC), University of Bologna, 40126 Bologna, Italy; 4 Gynecology and Obstetrics Unit, Department of Neuroscience, Reproductive Sciences and Dentistry, Federico II University of Naples, 80131 Naples, Italy

**Keywords:** Serous endometrial intraepithelial carcinoma (SEIC), Post-radiotherapy endometrial change, p53, Immunohistochemistry

## Abstract

Uteri from women undergoing chemoradiotherapy (CRT) may show reactive atypia which may mimic serous endometrial intraepithelial carcinoma (SEIC). We aimed to assess the prevalence and morphological/immunohistochemical features of post-radiotherapy serous-like endometrial changes (PoRSEC) in women undergone CRT for locally advanced cervical cancer, with a focus on the differential diagnosis with SEIC. Consecutive patients with locally advanced cervical cancer undergone CRT between 2011 and 2018 were reviewed. Endometrial histological specimens were assessed for the presence of PoRSEC. Twenty-two cases of SEIC were included for comparison. Immunohistochemistry for p53, p16, and Ki67 was performed. Out of 244 reviewed patients, 36 (14.7%) showed PoRSEC. The degree of nuclear atypia was similar between PoRSECs and SEIC. However, a papillary architecture with areas of confluent papillae was only observed in SEIC. SEIC cases showed a high mitotic activity as opposed to PoRSEC cases. The expression of p53 was aberrant in all SEICs but in none of the PoRSECs; however, 13/36 PoRSECs showed p53 positivity in most tumor cells, potentially mimicking a mutation pattern. A block-type p16 expression was observed in all SEICs and in 16/36 PoRSECs. Mean Ki67 expression was 26.9% in SEIC (range 5–70%) and 8.16% in PoRSEC (range 5–35%). While SEIC showed sharp morphological and immunohistochemical demarcation, PoRSEC were more heterogenous and merged imperceptibly with normal endometrium. In conclusion, PoRSEC may mimic SEIC both morphologically and immunohistochemically. However, a papillary architecture with cytological demarcation is typically observed in SEIC but not in PoRSEC.

**Supplementary Information:**

The online version contains supplementary material available at 10.1007/s00428-024-03818-4.

## Introduction

With the increasing use of concurrent chemoradiotherapy (CRT) plus brachytherapy as a standard of care in patients with locally advanced cervical carcinoma (LACC, stage IB2-IVA, FIGO staging classification, 2018) [1], the recognition of radiation-associated histological changes has become mandatory. Endocervical histological changes after radiotherapy have been reported. Indeed, endocervical cells could appear enlarged; with an increased nuclear-to-cytoplasm ratio, the cytoplasm could be eosinophilic or finely vacuolated and the nuclei could loss polarity and show prominent eosinophilic nucleoli [2]. CRT-related changes can also be observed in endometrial epithelial cells; these changes should be considered when assessing endometria undergone CRT-related as they could mimic serous intraepithelial carcinoma (SEIC). However, to the best of our knowledge, these changes have only been described in case reports or small case series [3, 4]. Moreover, the differential diagnosis between CRT-related endometrial changes and SEIC has never been systematically assessed.

On this account, the aim of this study was to assess the prevalence, morphological features, and immunophenotype of post-radiotherapy serous-like endometrial changes (PoRSEC) and the differential diagnosis with SEIC.

## Materials and methods

The study has been approved by our Institutional Review Board (IRB) n° IST DIPUSVSP-17–05-2134, and it complied with the Ethical Principles for Medical Research Involving Human Subjects according to the World Medical Association Declaration of Helsinki.

### Study population

All consecutive hysterectomy specimens (*n*. 244) of patients with locally advanced cervical carcinoma (LACC) managed by CT/RT plus brachytherapy followed by surgery at the Gynecologic Oncology Unit and Radiotherapy of the Catholic University in Rome (Italy), from January 2011 to December 2018, were included. PoRSEC was defined as the presence of an endometrial gland with an epithelium showing the following features: hobnail cells, clear or eosinophilic cytoplasm, nuclear enlargement, hyperchromasia, pleomorphism, and presence of nucleoli.

Twenty-two cases of SEIC were used to compare morphological and immunohistochemical features.

All cases were reviewed by four pathologists with expertise in gynecological pathology (G.F.Z., D.A., G.S., N.D.A., and A.T.).

### Immunohistochemistry

Immunohistochemistry was performed according to previously described methods [5] and involved p53 antibodies (clone Do-7), p16 (clone 6H12) (Leica Biosystems, Wetzlar, Germany), Ki67 (clone 30–9), Napsin A (clone MRQ-60, Roche Ventana), and p504s (clone 13H4, Dako).

Immunohistochemistry expressions were categorized according to previously described methods [5, 6]; in particular, p53 was defined as “aberrant” (moderate-to-strong positivity in > 80% of cells or complete absence or cytoplasmic), “wild-type (wt)-high” (positivity in 50–80% of cells with variable intensity), “wt-intermediate” (positivity in 5–50% of cells), and “wt-low” (positivity in < 5% of cells).

Ki67 was reported as the percentage of epithelial cells showing any nuclear staining.

P16 was defined as positive *block-type* if it showed a “strong and diffuse” nuclear and cytoplasmic staining, diffuse patchy if more than 50% of the cells were positive, low-patchy expression if the positive cells were 5–50%, and absent if < 5%.

The expression of the Napsin A and p504s was defined as negative, focal, and diffuse.

## Results

### Morphology

Among 244 reviewed uteri, PoRSECs were observed in 36 (14.7%) cases.

**Fig. 1:**
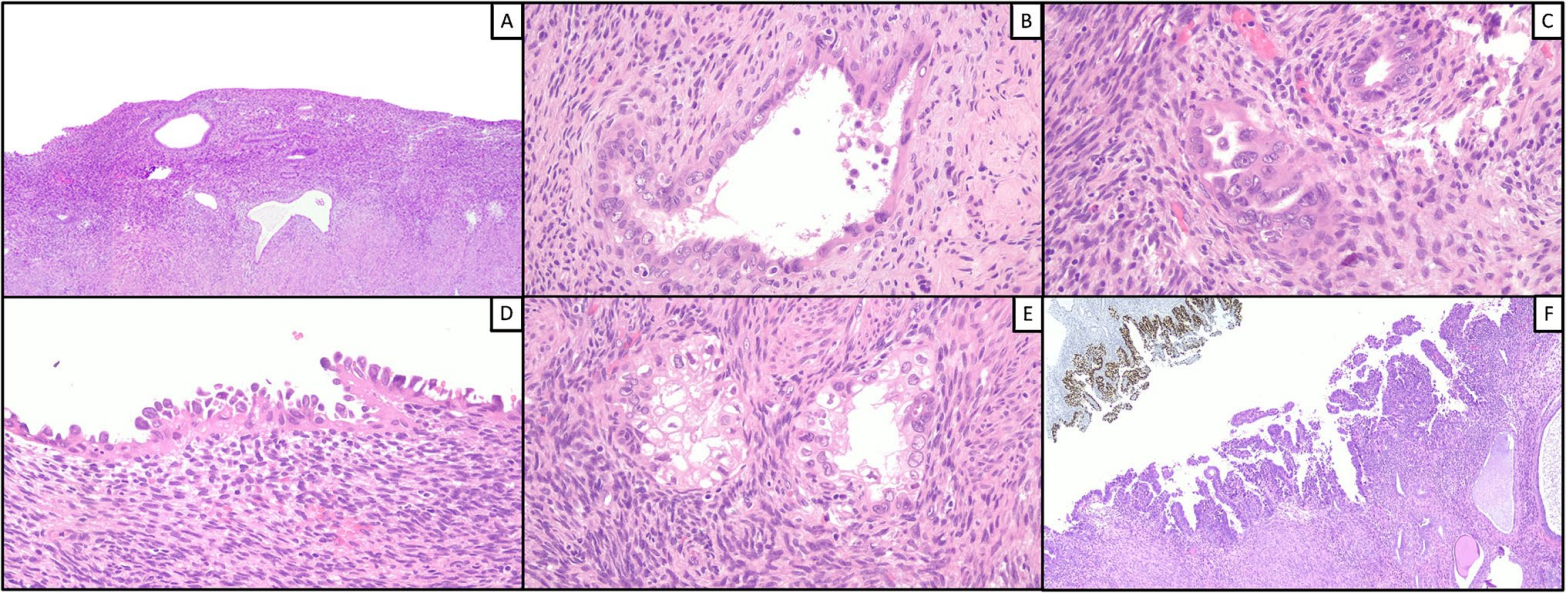
Morphological features of PoRSEC. SEIC-like glands that merged imperceptibly with more bland areas characterize endometria with PoRSEC (**A**). Nuclear pleomorphism and hyperchromasia with occasional nucleoli, frequent hobnail cells, eosinophilic cytoplasm, and clarification are the main features of PoRSEC (**B**, **C**, **D**, **E**). Note the simple papillae with areas of confluence and the sharp cytological demarcation between SEIC (with aberrant p53) and uninvolved endometrium (**F**)

Cell morphology was similar between PoRSEC and SEIC, as both showed nuclear pleomorphism and hyperchromasia with occasional nucleoli, frequent hobnail cells, and eosinophilic cytoplasm, sometimes with clarification. Regarding architecture, neither SEIC nor PoRSEC showed branching papillae or prominent glandular crowding; however, unlike PoRSECs, SEICs were at least focally characterized by simple papillae with areas of confluence. An evident mitotic activity was noted in SEIC cases but not in PoRSEC cases. A widespread involvement of the endometrium was observed in most PoRSEC cases (26/36, 72.2%) but also in a minority of SEIC cases (4/22, 18.2%). In all SEIC cases, there was a sharp cytological demarcation between SEIC and uninvolved endometrium; in PoRSEC cases, SEIC-like areas merged imperceptibly with more bland areas (Fig. 1).

### Immunohistochemistry

**Fig. 2:**
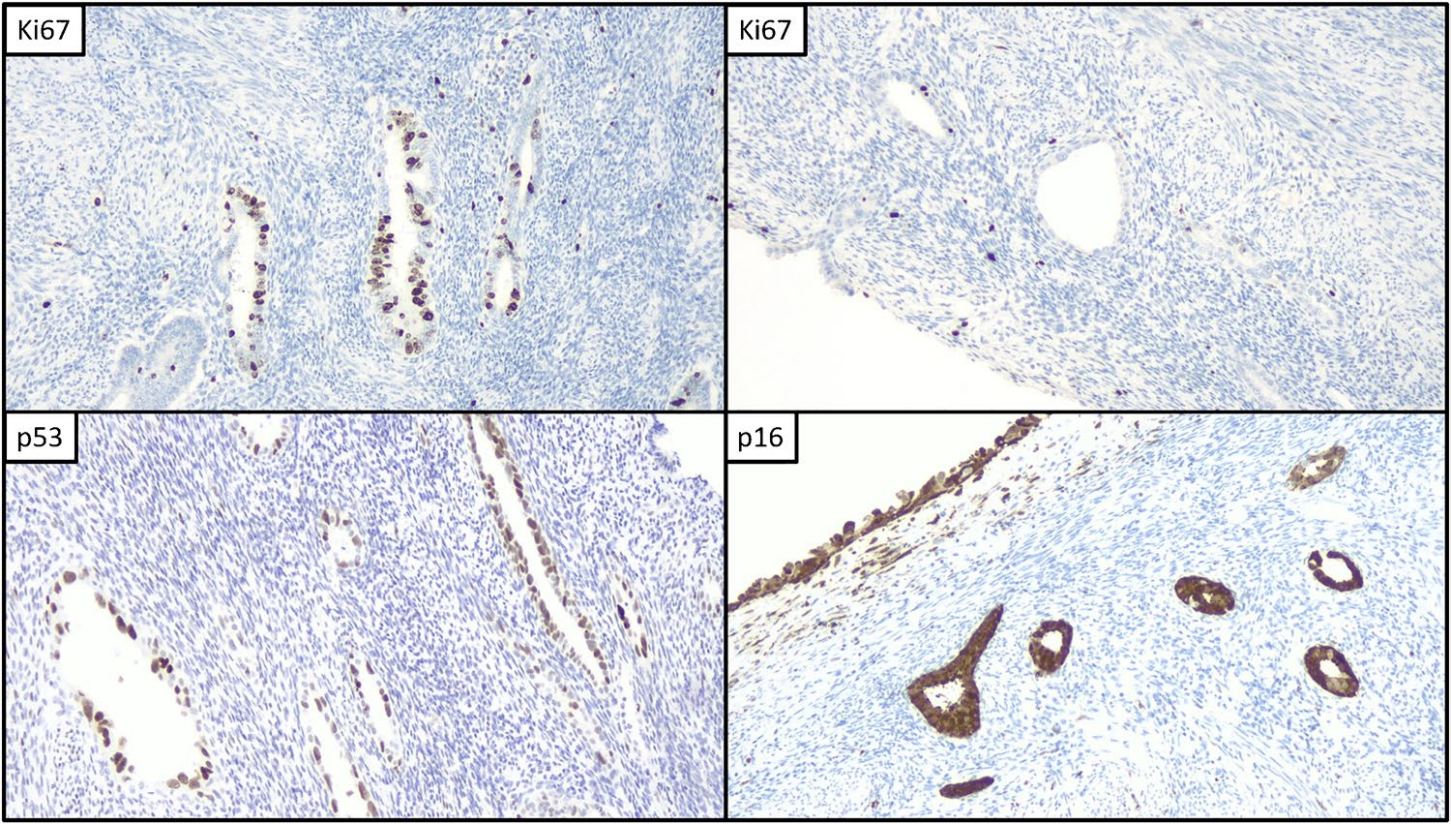
Immunohistochemical features of PoRSEC. Different range of Ki67, overexpression of p53, and block-type positivity of p16

**Fig. 3:**
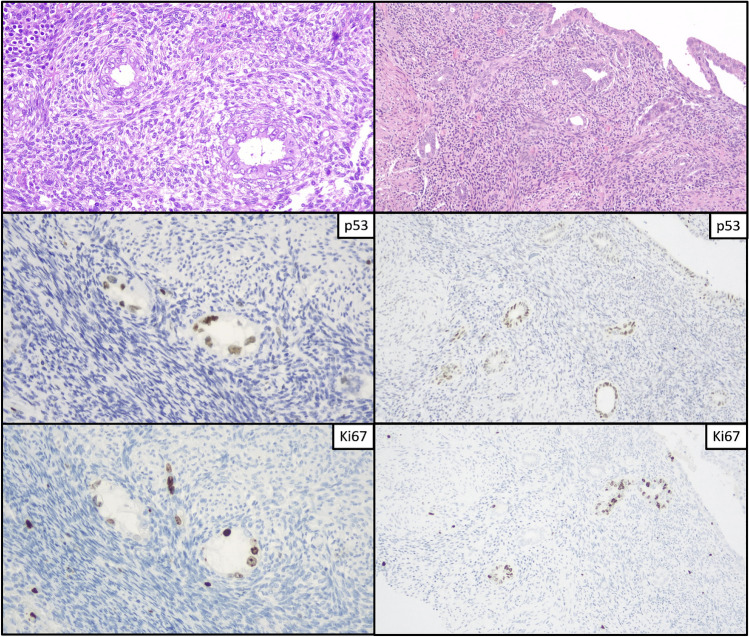
Morphological and immunohistochemical features of PoRSEC. Two cases with endometrial glands showing nuclear atypia and occasional nucleoli with high expression of p53 and low-to-high Ki67 index

All SEIC cases showed a mutant pattern of p53 (moderate-to-strong positivity in > 80% of cells) overexpression, while PoRSEC cases showed a wild-type expression, including a wt-low pattern in 6/36 (16.7%) cases, a wt-intermediate pattern in 17/36 (47.2%) cases, and a wt-high pattern in 13/36 (36.1%) cases. Ki67 index was higher than 10% in only a minority of PoRSEC (8/36, 22.2%), with the higher value being 35%. On the other hand, all but three SEIC cases (86.4%) had a Ki67 index ≥ 10%, with the higher value being 70%. The mean Ki67 index was 8.2% in PoRSEC and 26.6% in SEIC (Figs. 2, 3). P16 showed a *block-type* pattern in all cases of SEIC and in 16/36 (44.4%) PoRSEC cases; the remaining PoRSEC cases showed a diffuse-patchy expression (16/36, 44.4%), a low expression (3/36, 8.3%), or absent expression (1/36, 2.8%) (Table 1).

**Table 1:** Clinical, morphological, and immunohistochemical data

Diagnosis	PoRSEC	SEIC
N. patients	36	22
Age	50.2	67.3
Endometrial involvement		
*Focal*	10	18
*Diffuse*	26	4
Ki67 (mean)	8.20%	26.60%
p16		
*BT*	16	22
*DP*	16	0
*LP*	3	0
*Abs*	1	0
p53		
*Ab*	0	22
*H-wt*	13	0
*I-wt*	17	0
*L-wt*	6	0

p16—*BT* block type, *DP* diffuse patchy, *LP* low patchy, *Abs* absent. p53—*H-wt*-high wild type, *I-wt*-intermediate wild type, *L-wt*-low wild type, *Ab* aberrant

**Fig. 4:**
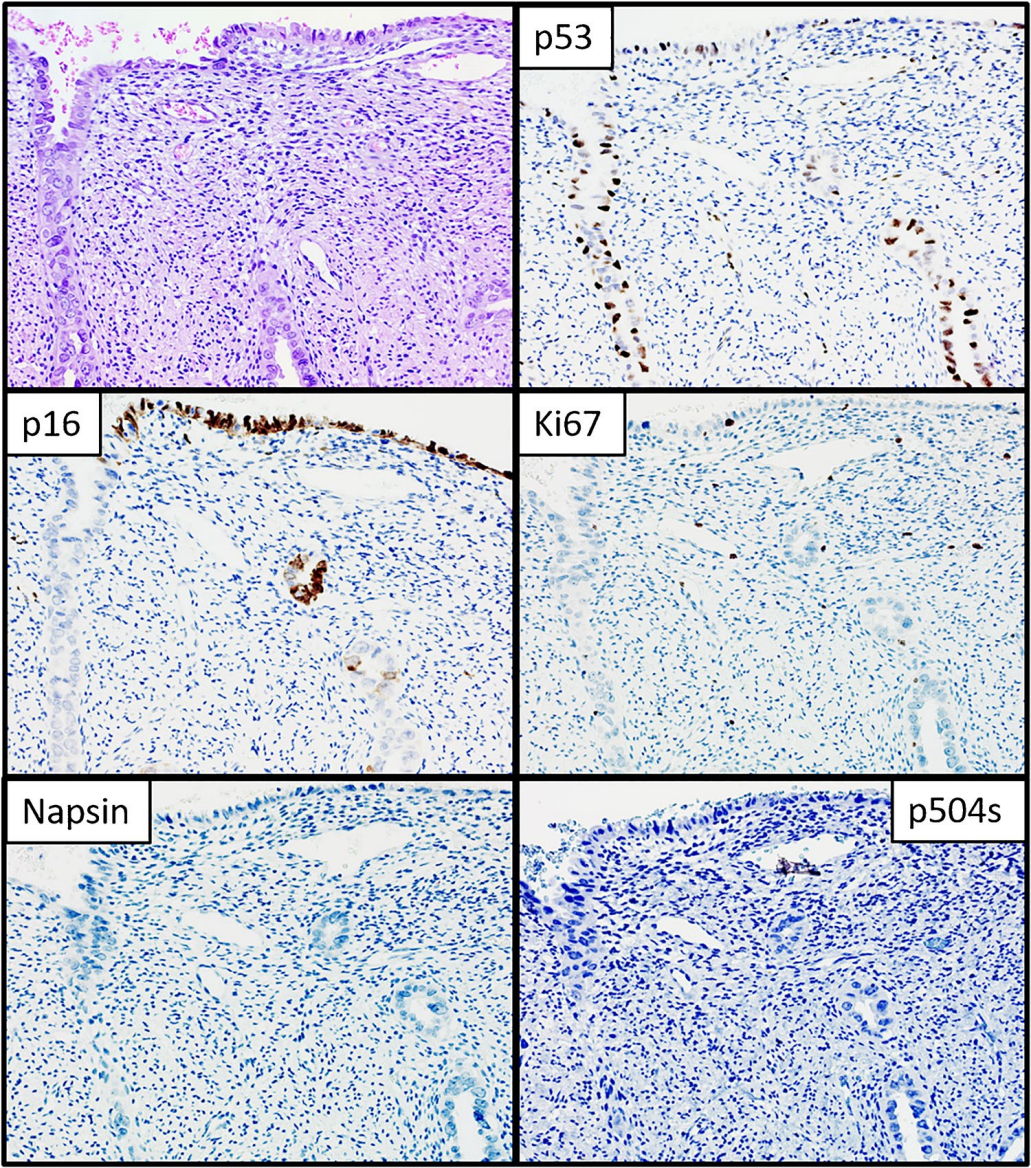
Morphological and immunohistochemical features of PoRSEC. A case with endometrial glands showing hobnail appearance with expression of p53 and p16 with a low Ki67 index. No expression of NapsinA and p504s was observed

Twenty-one cases with hobnail or clear cell appearance were tested for NapsinA and P504S and were all negative (Fig. 4).

## Discussion

This study showed that PoRSEC may show morphological and immunophenotypical overlap with SEIC. However, a papillary architecture with confluent papillae is observed in SEIC but not in PoRSEC. Moreover, SEIC shows cytological demarcation with normal endometrium, while PoRSEC appears as a diffuse change which merges imperceptibly with normal glands.

The distinction between reactive/regenerative atypia and true neoplastic atypia can be challenging. In the endometrium, reactive changes may raise the concern of several types of premalignant and malignant lesions, such as atypical hyperplasia, complex papillary proliferations, and SEIC [7–10]. Atypical endometrial hyperplasia and complex papillary proliferations are characterized by glandular crowding and branching papillae, respectively, which are typically absent in reactive changes [11, 12]. By contrast, SEIC may replace the epithelial lining of endometrial atrophic glands without affecting their arrangement. The typical feature of SEIC is the presence of high-grade nuclear atypia with striking pleomorphism [13]. Endometrial reactive changes can show an “SEIC-like” atypia, including nuclear enlargement and pleomorphism, nuclear clarification or hyperchromasia, evident nucleoli, eosinophilic or clear cytoplasm, and hobnail changes [9, 10]. This kind of atypia was also observed in our series of PoRSECs.

Our study was the first to systematically assess the morphological and immunophenotypical features of PoRSEC and the differential diagnosis with SEIC.

We found that, although the degree of atypia was similar between PoRSECs and SEICs, the former showed no evident mitotic activity. This finding is coexistent with those of our previous study, which showed no mitotic activity in endometrial metaplastic/reactive changes coexistent with cancer [5]. However, in our experience, mitotic figures can occasionally be found in reactive changes; this can be concerning, especially when the amount of examined tissue is scarce.

Although SEIC lacks branching papillae and prominent glandular crowding (as discussed above), it does show architecture complexity in the form of simple papillae which are areas of confluence, typically restricted to the surface endometrium, which was not observed in PoRSEC. Moreover, SEIC appeared as a circumscribed lesion, with a sharp cytological demarcation between the SEIC area and the background atrophic endometrium. On the other hand, PoRSECs extensively involved the endometrium with no abrupt transition between areas with high-grade atypia and areas with low-grade or no atypia.

Immunohistochemically, SEIC is characterized by a mutation-type p53 pattern; this reflects the presence of underlying *TP53* mutation and can also be observed in the earliest precursor of serous carcinoma, i.e., the so-called p53 signature [13]. Consistently, we observed a mutation-type p53 pattern in all included SEIC cases. As expected, all PoRSEC cases showed a p53-wt expression; however, more than one-third of PoRSEC cases had a “wt-high” pattern, that is, a p53 positivity in 50–80% of tumor cells. Before strict criteria to define a mutation-type pattern were defined, a wt-high pattern was often interpreted as aberrant [14]. However, even in the presence of strict criteria, the distinction between the two patterns can be difficult at times.

P16 and Ki67 are adjunctive markers in the diagnosis of SEIC. Indeed, SEIC characteristically shows a strong and diffuse (“block-type”) p16 expression and a high Ki67 expression (indicating a high proliferation index) [15]. A block-type p16 expression was observed in all SEIC cases and in almost half of PoRSEC cases. A similar finding was observed in metaplastic/reactive changes in our previous study and may constitute a further pitfall. Regarding Ki67, the mean value of SEIC was considerably higher than that of PoRSEC; however, the two groups showed a partial overlap, with the highest value among PoRSEC being 35% and the lowest value among SEIC being 5%.

Overall, PoRSEC showed a heterogeneous pattern of immunohistochemical markers, as opposed to SEIC. Therefore, SEIC shows both a morphological and immunohistochemical demarcation, which is generally absent in PoRSECs.

Finally, a complete absence of NapsinA and p504s staining could be useful to rule out a diagnosis of clear cell—EIN.

Remarkably, this study only included patients with LACC treated with CRT, in which PoRSEC can be expected. However, in our experience, PoRSEC can also be observed in benign uteri from patients who underwent CRT for other carcinomas. If the information regarding the previous CRT is missing, the pathologist might not consider the possibility of PoRSEC. It appears therefore necessary to recognize the crucial morphological and immunohistochemical features of PoRSEC, in order to avoid a serious misdiagnosis.

## Conclusion

Endometria from patients with LACC can show PoRSECs, which may morphologically and immunohistochemically mimic SEIC. Although the expression of p53, p16, and Ki67 is advisable for the differential diagnosis, there may be an overlap between SEIC and PoRSEC. In such a case, the presence of morphological and immunohistochemical demarcation, which is present in SEIC but not in PoRSECs, may be a useful aid.

## Supplementary Information

Below is the link to the electronic supplementary material.

Supplementary file1 (DOCX 15 KB)
