# Supplementary material for: Morphological and immunohistochemical evaluation in distinguishing post-radiotherapy serous-like endometrial change (PoRSEC) and serous endometrial intraepithelial carcinoma (SEIC)
Source: Virchows Arch. 2024 Jul 30;485(6):989–94. doi: 10.1007/s00428-024-03818-4 (PMC11666609; doi:10.1007/s00428-024-03818-4)
Supplement: Supplementary file 1 — Supplementary file1 (DOCX 15 KB) [file 428_2024_3818_MOESM1_ESM.docx]

| **ID Case** | **Diagnosis** | **Age** | **Endometrial involvement** | **Ki67 (%)** | **p16** | **p53** |
| --- | --- | --- | --- | --- | --- | --- |
| 1 | PoRSEC | 58 | Diffuse | 5 | BT | H-wt |
| 2 | PoRSEC | 76 | Focal | 5 | BT | H-wt |
| 3 | PoRSEC | 52 | Diffuse | 5 | BT | H-wt |
| 4 | PoRSEC | 52 | Diffuse | 5 | BT | H-wt |
| 5 | PoRSEC | 46 | Diffuse | 5 | BT | H-wt |
| 6 | PoRSEC | 37 | Diffuse | 5 | BT | H-wt |
| 7 | PoRSEC | 46 | Diffuse | 8 | BT | I-wt |
| 8 | PoRSEC | 49 | Diffuse | 8 | BT | I-wt |
| 9 | PoRSEC | 30 | Focal | 5 | BT | I-wt |
| 10 | PoRSEC | 61 | Focal | 5 | BT | I-wt |
| 11 | PoRSEC | 49 | Diffuse | 5 | BT | I-wt |
| 12 | PoRSEC | 61 | Diffuse | 5 | BT | I-wt |
| 13 | PoRSEC | 57 | Focal | 5 | BT | L-wt |
| 14 | PoRSEC | 39 | Focal | 5 | BT | L-wt |
| 15 | PoRSEC | 60 | Diffuse | 5 | BT | L-wt |
| 16 | PoRSEC | 36 | Diffuse | 5 | BT | L-wt |
| 17 | PoRSEC | 37 | Diffuse | 30 | DP | H-wt |
| 18 | PoRSEC | 44 | Diffuse | 20 | DP | H-wt |
| 19 | PoRSEC | 52 | Diffuse | 5 | DP | H-wt |
| 20 | PoRSEC | 33 | Diffuse | 8 | DP | H-wt |
| 21 | PoRSEC | 52 | Diffuse | 5 | DP | H-wt |
| 22 | PoRSEC | 73 | Diffuse | 35 | DP | I-wt |
| 23 | PoRSEC | 70 | Diffuse | 5 | DP | I-wt |
| 24 | PoRSEC | 64 | Diffuse | 10 | DP | I-wt |
| 25 | PoRSEC | 37 | Diffuse | 20 | DP | I-wt |
| 26 | PoRSEC | 40 | Diffuse | 10 | DP | I-wt |
| 27 | PoRSEC | 43 | Diffuse | 10 | DP | I-wt |
| 28 | PoRSEC | 74 | Diffuse | 5 | DP | I-wt |
| 29 | PoRSEC | 40 | Diffuse | 10 | DP | I-wt |
| 30 | PoRSEC | 33 | Diffuse | 5 | DP | I-wt |
| 31 | PoRSEC | 51 | Diffuse | 5 | DP | I-wt |
| 32 | PoRSEC | 54 | Focal | 5 | DP | L-wt |
| 33 | PoRSEC | 36 | Focal | 5 | LP | H-wt |
| 34 | PoRSEC | 58 | Focal | 5 | LP | I-wt |
| 35 | PoRSEC | 54 | Focal | 2 | LP | L-wt |
| 36 | PoRSEC | 56 | Focal | 8 | A | H-wt |
| 37 | SEIC | 70 | Focal | 20 | BT | Ab |
| 38 | SEIC | 70 | Focal | 40 | BT | Ab |
| 39 | SEIC | 57 | Focal | 20 | BT | Ab |
| 40 | SEIC | 61 | Focal | 15 | BT | Ab |
| 41 | SEIC | 71 | Focal | 30 | BT | Ab |
| 42 | SEIC | 80 | Diffuse | 25 | BT | Ab |
| 43 | SEIC | 75 | Focal | 5 | BT | Ab |
| 44 | SEIC | 68 | Focal | 5 | BT | Ab |
| 45 | SEIC | 61 | Diffuse | 30 | BT | Ab |
| 46 | SEIC | 54 | Focal | 20 | BT | Ab |
| 47 | SEIC | 68 | Focal | 10 | BT | Ab |
| 48 | SEIC | 80 | Diffuse | 70 | BT | Ab |
| 49 | SEIC | 69 | Diffuse | 25 | BT | Ab |
| 50 | SEIC | 59 | Focal | 30 | BT | Ab |
| 51 | SEIC | 77 | Focal | 35 | BT | Ab |
| 52 | SEIC | 73 | Focal | 35 | BT | Ab |
| 53 | SEIC | 65 | Focal | 25 | BT | Ab |
| 54 | SEIC | 76 | Focal | 25 | BT | Ab |
| 55 | SEIC | 53 | Focal | 20 | BT | Ab |
| 56 | SEIC | 74 | Focal | 5 | BT | Ab |
| 57 | SEIC | 48 | Focal | 70 | BT | Ab |
| 58 | SEIC | 71 | Focal | 25 | BT | Ab |
